# Mechanical texture profile of Hanwoo muscles as a function of heating temperatures

**DOI:** 10.1186/s40781-018-0181-9

**Published:** 2018-09-18

**Authors:** Ochirbat Chinzorig, Inho Hwang

**Affiliations:** Department of Animal Science and Biotechnology, Chonbuk National University, Jeonju, 561-756 Republic of Korea

## Abstract

**Background:**

Cooking temperature and consequently doneness of beef muscles are most important for the palatability and consumer acceptability. Current study assessed the response of mechanical texture of Hanwoo muscles as a function of cooking temperature at different ageing days. Six muscles (*Psoas major* (PM), *Longissimus thoracics* (LT), *Gluteus medius* (GM), *Semimembranosus* (SM), *Biceps femoris* (BF) and *Triceps brachii* (TB)) were collected from each 10 Hanwoo steers. Warner-Bratzler WB-shear force (WBSF) and texture profile analysis (TPA) texture profiles were determined after 3 or 21 days of chiller, and randomly assigned to four groups; non-cooked, cooked at 55, 70 or 85 °C.

**Results:**

Toughness of WBSF and TPA hardness of Hanwoo muscles were presence in the order of LT = PM = GM = SM < BF = TB (*p* < 0.001) for non-cooked raw muscle, and PM < LT = GM = SM < TB=BF (*p* < 0.001) for cooked meat aged for 3 days. WBSF linearly increased in 3 days aged meats after cooked at a higher temperature (*P* < 0.05). On the other hand, toughening of the muscles were significantly (*P* < 0.05) differed at various temperature when muscles were aged for 21 days. WBSF of PM and LT muscles were significantly increased at a higher cooking temperature, while other muscles (i.e., GM, SM, BF, TB) showed the lowest values at 70 °C. In the case of TPA hardness, the effect of cooking temperature was very less in the toughness of the muscle (*P* > 0.05).

**Conclusion:**

Taken together, these findings clearly showed that the toughness of the muscle highly depends and varies upon the temperature and ageing of the muscle. Moreover, the effect of cooking temperature was very limited on aged muscles. The results mirror the importance of cooking temperature for objective measurements which ultimately estimate sensory tenderness and other quality traits.

## Background

Consumer satisfaction and acceptance of beef steaks is greatly dependent on degree of doneness, as affected by culture and texture property [17]. A large number of previous studies have been reported a significant linkage between beef tenderness and degree of doneness [24, 26, 30] and also reported that the good quality of meat steaks had less juiciness and higher toughness. The results were most likely related to the hardening of myofibril component and shrinkage relation of collagen fibres [4], as well as the denaturation and/or solubilisation of the meat protein components during heating [14].

An early pioneer study [3] demonstrated that contributions of each myofibrillar or/and connective tissue components greatly relayed on intrinsic and extrinsic factors such as animal, muscle, pH and the cooking temperature to the structural strength of meat samples. More details are further confirmed by fact that muscles with higher collagen content was tenderized at 45–65 °C and toughened at 65–80 °C, while muscles with lower collagen was tenderized at 45–55 °C and toughened at above 55 °C [21]. It seems a fact that connective tissue makes the largest contribution to texture in raw meat while myofibers make the largest contribution to texture in cooked meat [1]. Similarly, based on the end-point cooking temperature for beef longissimus muscle, Yang et al. [31] noted connective tissue component appeared to be highly toughness up to 60 °C of cooking temperatures, while myofibrillar components became more important at cooking temperatures.

According the national consumer audit [20], Korean beef consumers preferred grilling and roasting of thin sliced steaks. This implies that end-point temperature of cooking, consequently doneness, is greatly important for the palatability and consumer acceptability of beef products. Average market turn-over of Hanwoo beef is approximately 14 days, while that has great variation from 1 to 30 days. Meat quality parameters such as intramuscular fat and collagen content are extremely great between Hanwoo steers and within Hanwoo muscles [27]. Given this reason, toughness is the most importance factor determining acceptability of cooked beef for various type of cooking methods [7]. Recently, Korean government has reviewed the beef grading system to consolidate texture of muscles. As far as we aware that there is no accessible date to understand texture profiles of non-cooked Hanwo muscles and their changes at various cooking temperature. Given that, the present study is designed to assess the response of mechanical texture of Hanwoo muscles as a function of cooking temperature including non-cooked raw meat at different ageing days.

## Results and discussion

### Texture profile of non-cooked meats for various muscle type

Prediction and/or estimation of texture traits for the cooked meats from not-cooked raw muscle is greatly important to design and optimizes processes, and ultimately to achieve certain textural characteristics. The current analysis focused on the effect of cooking on mechanical texture profile for various muscles. Objective mechanical measurements of non-cooked raw meat and cooked at 70 °C Hanwoo muscles after chiller aged for 3 days are showed in Table 1. At the first experimental design, these results were prime interesting because palatability of cooked beef is greatly influenced by heating process and more importantly that varies depending on muscle type [13]. Korea beef grading system includes ‘texture’ as a factor of carcass quality assessment [27]. Although magnitude of the texture factor for the final quality grading is currently very limited, given the updated next general of grading scheme, the factor appears to become very much significant for the final grading. Our results, at first glance, indicated that cooking process significantly (*P* < 0.05) increased WBSF and hardness and elevation of toughness by thermal denaturation was greatly affected by muscle type. This was not a surprising, but rather expected. Previous studies for European beef breeds suggested that toughening by cooking products were as a function of myofibril, connective and fat content [5].

**Table 1:** Least square means of WBSF and TPA texture profiles for six Hanwoo steer muscles of raw meat and cooked at 70 °C after aged for 3 days

Muscles^ǂ^	WBSF(kgf)		Hardness(kgf)^Ψ^		Springiness		Cohesiveness		Chewiness	
	Raw	Cooked	Raw	Cooked	Raw	Cooked	Raw	Cooked	Raw	Cooked
PM	2.87^bY^	3.99^cX^	2.47^cbY^	4.22^cX^	1.04^aX^	0.49^dY^	0.01	0.01^b^	0.16^aX^	0.00^cY^
LT	2.74^bY^	4.30^bX^	2.09^cY^	5.86^bX^	0.69^c^	0.74^cb^	0.01^Y^	0.02^abX^	0.04^b^	0.02^c^
GM	3.25^bY^	4.56^bX^	2.91^cbY^	6.13^abX^	0.93^abcX^	0.70^cY^	0.01	0.01^b^	0.1^ab^	0.08^bc^
SM	4.01^bY^	5.06^bX^	3.19^bY^	6.50^abX^	0.98^ab^	0.85^b^	0.01^Y^	0.01^aX^	0.1^ab^	0.24^b^
BF	6.07^aY^	7.59^aX^	4.42^aY^	7.71^aX^	1.06^a^	1.18^a^	0.01^Y^	0.02^aX^	0.14^aY^	0.48^aX^
TB	7.64^aY^	8.46^aX^	6.42^aY^	8.96^abX^	0.76^bc^	0.89^bc^	0.01	0.01^b^	0.09^ab^	0.12^bc^
SEM	0.92	0.29	0.32	0.51	0.09	0.07	0.001	0.002	0.02	0.06
F value (Muscle df 5/53; Treatment df 1/107)										
Muscle	6.85^***^	8.46^***^	9.56^***^	5.36^***^	2.84^*^	11.6^***^	0.61	3.92^**^	2.7^*^	10.5^***^
Treat.	7.50^**^		115.32^***^		4.38^*^		2.27		4.33^*^	

^ǂ^PM, *Psoas major;* LT, *Longissimus thoracis;* GM, *Gluteus medius;* SM, *Semimembranosus;* BF, *Biceps femoris;* TB, *Triceps brachi.* ^a-c^, means within each column with different superscripts are significantly different

^X, Y^means within each row with different superscripts are significantly different

^***^p < 0.001, ^**^p < 0.01, ^*^p < 0.05. df, degrees of freedom

^Ψ^Hardness: the peak force that occurs during the first compression, Springiness = Length 2/Length 1; Cohesiveness = Area 2/Area 1; Chewiness = Hardness x Springiness x Cohesiveness (Fig. 2)

Values of WBSF of the non-cooked raw muscle were showed in the order of LT = PM = GM = SM < BF = TB (*p* < 0.001), and hardness for the raw samples showed similar trends with a simple correlation between these two measurements were 0.97. For the cooked muscles at 70 °C after 3 days of chiller ageing, WBSF values were presence in the order of PM < LT = GM = SM < TB=BF (*p* < 0.001) with a similar tendency of hardness values (*r* = 0.87). The tenderness of Hanwoo muscle obtained in the present study are correlated with previous reports studied in Hanwoo muscles [15] and Angus and Brahman breeds [19]. In these observation and comparisons, there are two important findings should be taken for count is that the magnitude of the toughening by heating process was more great at the hardness assessment and WBSF. The second findings is the in toughness by the heating process was more significant (*P* < 0.05) for tougher muscles. The results implied that TPA hardness of raw muscle is more predictable measurement for cooked meat, especially for tougher muscles. Magnitude of toughening assessed by TPA hardness during cooking was 1.75, 3.77, 3.22, 3.31, 3.29, and 2.54 kg for PM, LT, GM, SM, BF and TB, respectively. In mean time, toughening of WBSF were 1.12, 1.56, 1.31, 1.05, 1.52, and 0.82 for PM, LT, GM, SM, BF and TB, respectively.

Previous studies have reported that WBSF measurements differed between raw and cooked meats [25]. The present study showed that WBSF of raw meat is mainly reflecting background or collagen toughness, whereas that for cooked meat largely reflected myofibrillar toughness. More recently Listrat et al. [16] stated that WBSF for raw meat is highly correlated with collagen content and the relationship for cooked meat highly depends the muscle type because of the relationship between the thermal solubility and cross-linking level of collagen.

In contrarily, we are more interested in the responses of each muscle to the cooking process in terms of toughening during the data mining process. As previously mentioned WBSF of raw and cooked meats were presence in the order of LT = PM = GM = SM < BF = TB and PM < LT = GM = SM < TB=BF, respectively. The interesting fact in the result showed that LT is the most tender for non-cooked state, but cooked PM muscle became significantly (*P* < 0.05) more tender than LT. In other words, non-cooked samples of WBSF for LT, PM, GM and SM did not differ, while cooked samples of the same measurement for LT, GM, SM was identical.

**Fig. 1:**
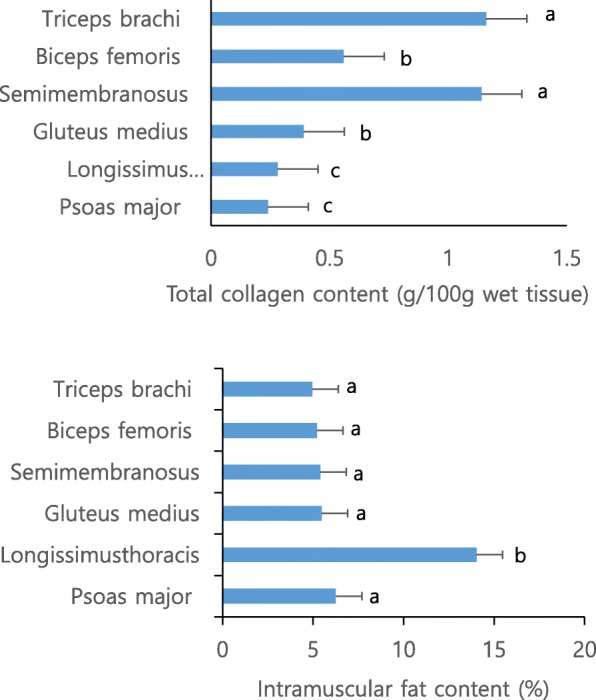
Least square means of total collagen content (top) and intramuscular fat content (bottom) six Hanwoo muscles. Bars indicates standard deviation. Letters within a same figure with a same letter did not differ significantly (*P* > 0.05)

Figure 1 shows the total collagen and intramuscular fats for the six muscles. The particular difference between LT and PM is intramuscular fat content (*P* < 0.05) with similar total collagen content. The current data for both muscles likely indicates that amount of intramuscular fat is more important factor of meat texture for cooked meats followed by raw meat. Although total collagen BF is greatly (*P* < 0.05) lower than TB and SM in the current study and BF is well known as containing high amount of collagen [28] (Weston et al., 2002). The simple comparison of texture of cooked and raw meat muscles, the non-cooked muscles showed that the texture of muscle can be altered by cooking process, and should be carefully taken before cooking process. The results of the present study can be supported by previous similar reports [16]. The study indicated that thermal solubility, cross-linking level of collagen and meat shear force varies based on the muscle type and cooking conditions.

### Interaction between cooking temperature and muscle type on texture profile for different muscles

It has been well documented that toughening of muscle tissue during cooking has at least three successive steps; where initial toughness up to around 50 °C ascribed to myofibrillar denaturation, and second toughening between 50 and 60 °C was related to collagen denaturation [18]. Furthermore, more detailed temperature kinetic of myofibril components are also reported for α-actinin (50 °C), myosin (55 °C), actin (70–80 °C), titin (73 °C), tropomyosin and troponin (over 80 °C) and nebulin survived upto 80 °C [22] (Palka and Henry, 1999).

In the current study, response of mechanical texture to various cooking temperature at 55 (rare), 70 (medium) and 85 °C (high) for various Hanwoo muscle at different ageing time (Table 2). However, the mechanical tenderness is highly different with sensory tenderness of the muscle [29]. The results indicated that WBSF was not a good predictor of tougher muscles and also revealed that WBSF linearly increased as meats aged for 3 days were cooked at a higher temperature. On the other hand, TPA hardness showed significantly (*P* < 0.05) lower values for 70 °C samples of BF and TB. The result was not completely correlated with previous reports, but the tenderness of the muscle was likely related to TPA hardness measurement for beef muscles. And the high collagen content as hardness was also greatly influenced by intramuscular fat content in beef *longissimus* muscles [23].

**Table 2:** Least square means of WBSF and TPA texture profile as a function of muscle and cooking temperature at different ageing days of Hanwoo steer muscles

Muscles^*ǂ*^	Cooking temp	WBSF(kg)		Hardness(kg) ^Ψ^		Springiness		Cohesiveness		Chewiness	
		3d	21d	3d	21d	3d	21d	3d	21d	3d	21d
*Psoas major*	55 °C	3.11	2.93^ab^	3.46^b^	3.71	0.82^a^	0.48^b^	0.01	0.01^b^	-0.02^ab^	−0.04
	70 °C	3.99	2.64^b^	4.22^ab^	4.08	0.49^b^	0.41^b^	0.01	0.01^b^	0.005^aX^	−0.05^Y^
	85 °C	4.51	3.40^a^	5.66^a^	4.41^a^	0.65^ab^	0.86^a^	0.02	0.03^a^	−0.10^b^	−0.1
*Longissimus thoracis*	55 °C	2.70^c^	2.59^b^	5.55	4.57	0.67	0.73	0.01^b^	0.01^b^	−0.02^a^	0.06^a^
	70 °C	4.30^b^	2.63^b^	5.74	5.47	0.74	0.69	0.02^b^	0.01^b^	0.02^a^	0.06^a^
	85 °C	5.06^a^	3.47^a^	5.86	4.92	0.84	0.79	0.04^a^	0.04^a^	−0.19^b^	−0.18^b^
*Gluteus medius*	55 °C	3.68^b^	3.73^b^	6.32	5.06	0.73	0.89	0.01^b^	0.01^b^	0.07^a^	0.11^a^
	70 °C	4.56^ab^	3.30^b^	6.13	5.72	0.7	0.65	0.01^b^	0.02^b^	0.08^a^	0.04^a^
	85 °C	5.09^a^	3.88^a^	5.52	6.01	0.79	0.96	0.03^a^	0.04^a^	−0.35^b^	−0.19^b^
*Semimembranosus*	55 °C	4.39^b^	3.65^b^	6.17^b^	6.33	0.94	0.99	0.01^b^	0.01^b^	0.16	0.27
	70 °C	5.06^b^	3.29^b^	7.05^ab^	6.68	0.92	0.83	0.02^b^	0.01^b^	0.21	0.09
	85 °C	5.84^a^	3.92^a^	8.49^a^	6.5	0.98	0.96	0.03^a^	0.04^a^	0.03	0.08
*Biceps femoris*	55 °C	5.96^b^	6.22^a^	8.12^a^	6.89^b^	0.96	1.05	0.01^b^	0.01^b^	0.19	0.29
	70 °C	7.59^a^	4.79^b^	7.71^b^	7.55^b^	1.18^X^	0.97^Y^	0.02^b^	0.02^b^	0.48^X^	0.13^Y^
	85 °C	7.93^a^	5.54^a^	8.69^a^	7.87^a^	1.24	1.28	0.04^a^	0.04^a^	0.46	0.36
*Triceps brachi*	55 °C	8.34	7.11^a^	9.56^a^	5.34^b^	1.01	0.77	0.01^b^	0.01^b^	0.29	0.11
	70 °C	8.46	4.00^b^	8.96^b^	6.7^b^	0.89	0.97	0.01^b^	0.02^b^	0.12	0.09
	85 °C	8	6.73^a^	9.32^a^	7.02^a^	1.24	1.14	0.04^a^	0.05^a^	0.58	−0.11
SEM		0.18	0.18	0.27	0.19	0.04	0.05	0.001	0.002	0.05	0.04
F value											
Muscle		23.63^***^	17.97^***^	18.54^***^	21.45^***^	9.07^***^	7.53^***^	2.81^*^	0.99	8.64^***^	4.23^***^
Temperature		4.97^**^	10.75^***^	1.27	5.44^**^	2.45	7.21^***^	52.7^***^	77.9^***^	0.72	3.45^*^
Muscle*Temperature		4.63^***^	3.25^***^	1.59	0.45	0.95	0.83	1.4	0.42	2.22^*^	0.96

^*ǂ*^PM*, Psoas major;* LT, *Longissimus thoracis;* GM, *Gluteus medius;* SM, *Semimembranosus;* BF, *Biceps femoris;* TB, *Triceps brachi;*

^a-c^means within each column with different superscripts are significantly different

^X, Y^means within each row with different superscripts are significantly different

^***^p < 0.001, ^**^p < 0.01, ^*^p < 0.05

df, degrees of freedom

**Fig. 2:**
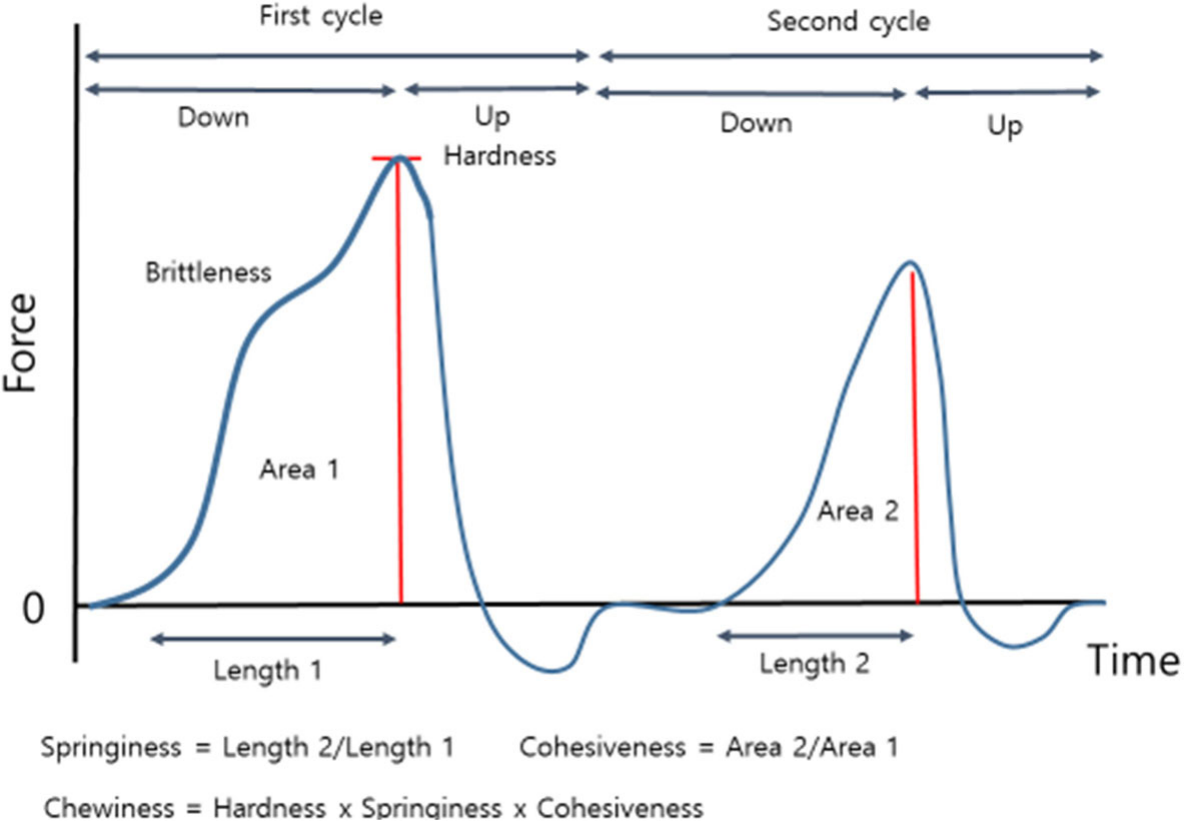
Schematic illustration of texture profile analysis (TPA) and its derivative measurements adopted in the current study

^Ψ^Hardness: the peak force that occurs during the first compression, Springiness = Length 2/Length 1; Cohesiveness = Area 2/Area 1; Chewiness = Hardness x Springiness x Cohesiveness (Fig. 2)

More surprisingly in the current data, the effect of cooking temperature was significantly differed in aged meat. WBSF of PM and LT muscles are significantly increased at a higher cooking temperature, while other muscles (i.e., GM, SM, BF, TB) showed the lowest values at 70 °C. In the case of TPA hardness, cooking temperature had a limited effects on toughness. LT, GM, SM showed an identical values for various cooking temperature, while BF and TB showed significantly (*P* < 0.05) higher values during high temperature cooking. Taken together, the results indicated that the effect of cooking temperature on toughness process gradually decreased as ageing time and increased for most type of muscles. Proteolytic process and tenderization during chiller ageing is well documented [30]. Given the fact, it was clearly understand that cooking temperature more significantly affects the connective tissue components, because the toughness of chiller aged meat are mostly attributed to background toughness and collagen component [12].

Another noticeable observation in the current study was that response of PM and LT muscle to the aging x cooking temperature interactions was very clear (*P* < 0.05) for WBSF. This was interesting point, as two muscles has completely different intramuscular fat content (6.2% for PM and 14.0 for LT) with similar total collagen content (0.24 and 0.28 g/100 g for PM and LT, respectively) (Fig. 1). Toughness of PM muscle aged for 3 days numerically (but not significant) linearly increased at a higher cooking temperature, while shear force for LT increased significantly (*P* < 0.05) as temperature increased from 2.70, 4.30 to 5.06 kg for 55, 70 and 85 °C. The tendency of PM and LT muscles aged for 21 days was also similar with 3 day ones. This likely mirrors that intramuscular content affects the effects of cooking temperature on toughness, although the mechanisms are not clearly explainable, because TPA hardness of LT was not influenced by cooking temperature at both day 3 and 21.

Springiness is defined as ratio of the time duration of force input during the second to that during the first compression and cohesiveness is defined as the ratio of positive force area during the second to that the fist compression cycle [8]. Early studies indicated that hardness accounted approximately 40.6 and 45.7 in tenderness and overall palatability [6]. Furthermore, they also noted that TPA springiness chewiness were highly related to intramuscular fat content and consequently tenderness and juiciness. To elucidate our experimental design which examined texture profiles as a function of heating temperature type at different ageing days, springiness, cohesiveness and chewiness were also determined, and the data was extensive explored during data mining process. Very limited information was obtained from the measurements, although springiness of meat is probably associated to fibre swelling and diameter [22]. Not all, but it should be noted that there was a tendency that ageing decreased springiness, while increased chewiness.

## Conclusion

Anticipation of cooked texture from non-cooked raw muscle has become more import from the consumer aspects of meat choice and satisfaction. The current data indicated that magnitude of thermal toughening greatly varied between beef muscles where hardening of muscle samples by heating was more obvious for tougher meat (i.e., BF, SM, GM, BF). Particularly. This implied that toughness of raw meat is not always a good predictor of cooked meat. Their results indicated that WBSF was not a good predictor of tougher muscles. Our results overall revealed that WBSF linearly increased as meats aged for 3 days were cooked at a higher temperature. On the other hand, interestingly, muscles aged for 21 days showed completely different response to the cooking temperatures where the temperature was very limited for most muscle types. Collectively, the current data indicated that estimation of meat texture from raw material to cooked meats varies depending on muscle type and its interaction with chiller ageing day. In addition, the results mirror the importance of cooking temperature for objective measurements which ultimately estimate sensory tenderness and other quality traits.

## Methods

### Sampling and experimental design

Ten (10) Hanwoo steer were sampled from a commercial feeding population and slaughtered at a commercial abattoir at a same day. An average age, hot carcass weight and backfat thickness were 28 months, 422 kg and 7.4 mm, respectively. Animals were transferred to approximately 50 km the day before slaughter and lairage over night with free access to water. The day after slaughter, six muscles (*Psoas major* (PM), *Longissimus thoracics* (LT), *Gluteus medius* (GM), *Semimembranosus* (SM), *Biceps femoris* (BF) and *Triceps brachii* (TB)) were taken from right side of carcass and used for analysis. Principle sampling and treatment design was 2 × 4 block design where 2 ageing length (3 or 21 days) and 4 end-point cooking temperature (non-cooked, 55, 70 or 85 °C). Each muscle divided into two potions, vacuum packaged, and randomly assigned for the two ageing groups at 4 °C. Objective texture profiles were assessed on fresh sample and the remaining tissue samples for stores at − 20 °C for chemical analysis.

### Objective texture measurements

Warner-Bratzler shear force (WBSF) and the texture profile analysis (TPA) were determined by cutting and pressing probes with an Instron Universal Testing Machine (Model 3342, USA) using shearing and compression devices [2, 4, 9, 10] (Bouton et al., 1972; 1974, Gupta et al., 2007; Herrero et al., 2007). Sample blocks were cooked at 55, 70, or 85 °C in polyethylene bags for 60 min in an 80 L pre-heated water bath (BS-21, Jeiotech Co., Korea). Starting temperature of the cooking blocks were 4 °C and the core temperature of samples was monitored using a copper–constant thermocouple attached to a thermos recorder (Model TR-71 U, T&D Corporaion, Japan). The cooked blocks were cooled in running cold tap water (approximately 18 °C) for 30 min.

For the WBSF measurement, six strips with average diameter of 0.5 in. and a length of at least 2 cm were removed parallel to the muscle fibre direction of each raw and cooked sample. Samples were sheared perpendicular to the fiber orientation using a V-shaped blade, at a crosshead speed of 400 mm/min and a 40 kgf load cell.

Following sampling for shear force, two wedges of 5 mm to 15 mm thickness were cut parallel to the muscle fibre direction to determine compression. Samples were placed by fitting the sloped wedge faces between the base plate and compression plunger (0.63 cm diameter), 10 mm apart. The measurement was expressed as kilograms-force (Kgf) which is required to drive the plunger at 50 mm/min, two cycles of 0.8 cm into the wedge giving a 20% compression ratio. The variables were evaluated for hardness (maximum force required to the first compress the samples in kg), cohesiveness (area2/area1; the ratio of positive force area during the second to that the fist compression cycle), springiness (length2/length1; ratio of the time duration of force input during the second to that during the first compression) and chewiness (hardness x springiness x cohesiveness; multiply hardness, springiness and cohesiveness) (Fig. 2).

### Total collagen contents and intramuscular fat (IMF)

The total collagen content in samples was determined after 16 h hydrolysis of 2 g of meat with 7 NH_2_SO_4_ at 105 °C using modified colorimetric method. Hydrolysate was diluted with 500 mL of distilled water in Erlenmeyer flask. Diluted filtrate (2 mL) was taken and mixed with chloramine T solution in a test tube and left for 20 min at room temperature. After adding 4-dimethyl-aminobenzaldehyde solution, the mixture was heated at 60 °C for 15 min. The absorbance of samples and hydroxyproline standards was determined at 558 nm using spectrophotometer. For heat stable (or insoluble) collagen content, homogenized meat sample was heated in a 77 °C water bath for 70 min in a 3 times dilution of Ringer’s solution [11], followed by centrifugation, residual fractions were hydrolyzed in 7 N H_2_SO_4_ for 16 h at 105 °C. After neutralization, the hydroxyproline content of hydrolyzate was determined according to the procedure outlined by Hill [11]. The amount of hydroxyproline content was determined from a standard curve and converted to the collagen content with a factor of 7.14.

IMF was determined by the Soxhylet method in triplicate following Ji et al. (2010). 5 g of sample tissue and 1.5 g of sea sand were put in the cylinder type paper and mixed together. The fat samples were dried at 102 °C for 5 h and fats were extracted by petroleum ether at 100 °C for 6 h and petroleum was evaporated using a heating mantle by heating the extract in a dry oven at 102 °C for 1 h. IMF was calculated by percentage of extracting fat weight and sample weight.

### Statistical analysis

Two separate analysis was performed. The first analysis compared non-cooked raw sample and cooking at 70 °C for muscles aged for 3 days in that muscle, cooking and their interactions were main effects with animals were random effects. The second analysis examined the effects of cooking temperature (non-cooked, 55, 70, 85 °C) for different muscle types in that muscle, cooking temperature and their interaction (if any) were main effects with animals were random effects. Ageing day was not included in the main model, and thus samples aged 3 or 21 days were assessed independently. At the initial data mining models, the ageing effects and their interactions with cooking temperature and muscles were tested and significantly effects were observed, but finally we decided to take cooking temperature and muscle as a main effect for each samples of day 3 and 21 days. Least square means were calculated by a general linear model as a function of ageing day and cooking method using a general linear model and significant difference were assessed the Duncan’s multiple-range test at *p* ≤ 0.05 (SAS, SAS Institute Ver. 9.3, Cary, NC, USA).
